# The Genetic Basis of Toxin Biosynthesis in Dinoflagellates

**DOI:** 10.3390/microorganisms7080222

**Published:** 2019-07-29

**Authors:** Arjun Verma, Abanti Barua, Rendy Ruvindy, Henna Savela, Penelope A. Ajani, Shauna A. Murray

**Affiliations:** 1 Climate Change Cluster, University of Technology Sydney, Sydney 2007, Australia; 2 Department of Microbiology, Noakhali Science and Technology University, Chittagong 3814, Bangladesh; 3 Finnish Environment Institute, Marine Research Centre, 00790 Helsinki, Finland

**Keywords:** dinoflagellates, toxins, transcriptomics, polyketides, alkaloids

## Abstract

In marine ecosystems, dinoflagellates can become highly abundant and even dominant at times, despite their comparatively slow growth rates. One factor that may play a role in their ecological success is the production of complex secondary metabolite compounds that can have anti-predator, allelopathic, or other toxic effects on marine organisms, and also cause seafood poisoning in humans. Our knowledge about the genes involved in toxin biosynthesis in dinoflagellates is currently limited due to the complex genomic features of these organisms. Most recently, the sequencing of dinoflagellate transcriptomes has provided us with valuable insights into the biosynthesis of polyketide and alkaloid-based toxin molecules in dinoflagellate species. This review synthesizes the recent progress that has been made in understanding the evolution, biosynthetic pathways, and gene regulation in dinoflagellates with the aid of transcriptomic and other molecular genetic tools, and provides a pathway for future studies of dinoflagellates in this exciting omics era.

## 1. Introduction

Marine microbial eukaryotes are a diverse group of organisms comprising lineages that differ widely in their evolutionary histories, ecological niches, growth requirements, and nutritional strategies [1,2,3,4,5]. Among marine microbial eukaryotes (protists), dinoflagellates are of immense ecological and evolutionary significance [3,6,7,8]. Recent studies on protist species richness in the world’s oceans using genetic tools, such as metabarcoding, have shown that approximately half of 18S rDNA richness is made up of dinoflagellate sequences [9,10]. This widespread diversity is due to their ability to adapt to a wide variety of ecological niches, largely depending on their complex survival strategies (such as photoautotrophy, symbiosis, mixotrophy, and heterotrophy) [11,12]. They possess significant variability in morphology, pigment composition, and photosynthetic activity along with a broad spectrum of biological activities that serves various ecological niches. Their occupation of such diverse environments can be witnessed in their fossil records that date back several hundred million years [3,4,11].

**Figure 1:**
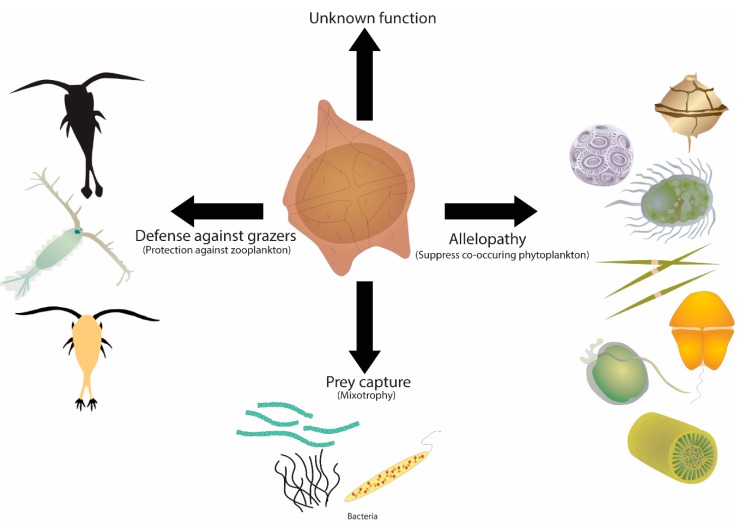
Functional role of toxins produced by dinoflagellates. Schematic representing various biological roles played by toxins for their producing organisms. Photo credit: Diana Kleine, Dieter Tracey, Ian Hewson, Jane Hawkey, Jane Thomas and Tracey Saxby, Integration and Application Network, University of Maryland Center for Environmental Science (ian.umces.edu/imagelibrary/).

Dinoflagellates are relatively inefficient at nutrient uptake, and exhibit slower growth rates compared to other protists such as chlorophytes, haptophytes, and diatoms [13,14]. Despite being poor competitors within their ecosystems, they can at times proliferate in large abundances, causing harmful algal blooms (HABs) [15,16]. Only about 2% of algal species have been reported as producing harmful blooms, of which 75% are dinoflagellates [14]. HABs are naturally occurring phenomena, however there is evidence that the frequency, geographic range, and intensity of these occurrences have increased over the past 30 years [17,18,19,20]. While the causes and consequences of HABs have been studied extensively, the production of toxic compounds by the culprit species and their interactions with co-occurring phytoplankton and potential predators remain to be fully understood [21,22]. These compounds display a wide variety of biological activity and may play distinct roles for producing organisms. One example of this is that the karlotoxins are produced by *Karlodinium veneficum* that may inhibit the growth of co-occurring plankton and immobilize potential prey species in close proximity [23,24]. Another example are the toxins produced by *Alexandrium* spp. that may play a role in grazing deterrence, with species-specific impacts found on copepods such as reduced grazing, reduced fecundity and delayed development [25,26,27,28,29]. Brevetoxins produced by *Karenia brevis* is also known to promote the survival of dinoflagellates by affecting grazer behavior [30,31,32]. Despite these studies, whether they are other as-yet-unidentified cellular functions of these compounds is currently unknown [32] (Figure 1).

Toxins produced during HABs can have widespread impacts on fisheries and aquaculture industries worldwide [17,33,34]. The most common effects of HABs are poisoning events, resulting in the deaths of marine organisms, and the accumulation of toxins in the marine food web, which may lead to eventual human poisoning via the consumption of contaminated seafood [35,36]. It is estimated that up to 60,000 human intoxications occur per year worldwide, with an overall mortality of approx. 1.5% [37,38]. The potential harm caused to commercially produced seafood by these marine toxins, and the associated human health risks are a concern for seafood safety regulatory bodies worldwide [39]. The greatest number of HAB related human health impacts have occurred due to toxic compounds that are classified as; saxitoxin (STX) and its analogues causing paralytic shellfish poisoning (PSP); brevetoxins (BTXs) and analogues causing neurotoxic shellfish poisoning (NSP); ciguatoxins (CTXs) and related compounds resulting in ciguatera fish poisoning (CFP); and okadaic acid (OA) and the dinophysis toxins (DTXs) causing diarrheic shellfish poisoning (DSP) [35,38]. Besides these well-known toxic compounds and their related illnesses, several new poisoning syndromes have recently appeared due to dinoflagellate toxins, such as azaspiracids (AZAs), yessotoxins (YTXs), and palytoxins (PLTXs) [38,40]. Approximately 20,000 marine natural products, with unique size, complexity, and biosynthetic pathways, have been discovered over the past 50 years, of which dinoflagellate toxins represent only a minor fraction, with many more that remain to be described [41]. Over the past 15 years, the first studies have begun to examine the genetic basis of bioactive compounds produced by algae, particularly the toxins synthesized by marine dinoflagellates. These studies have been challenging, largely due to the large size and the distinctive organization of dinoflagellate genomes compared to that of other eukaryotes [5,6].

## 2. Dinoflagellate Genomics

Dinoflagellate genomes are generally larger than the genomes of most other protists, ranging from 3–250 picograms of DNA per haploid genome, equating to around 1.2–112 × 10^9^ base pairs [42]. This genetic material is encoded in 24–220 nuclear chromosomes that are permanently condensed in a liquid crystalline state throughout the cell cycle, attached to the nuclear envelope and lacking nucleosomal heterochromatin [43,44,45]. Instead of nucleosomes, dinoflagellate DNA is associated with histone like proteins (HLPs) that are similar to bacterial DNA binding proteins (HU proteins) [46,47,48]. Dinoflagellate genomes have a generally low protein content (10:1 DNA: protein in the chromatin) and their histones exhibit frequent losses of key residues that are highly conserved among all other eukaryotes [49,50,51]. These HLPs are expressed at low levels and play a limited role in chromatin packaging [50,52]. This role of chromosomal organization is seemingly filled by viral-derived proteins called dinoflagellate-viral-nucleoproteins (DVNPs) that were potentially transferred from viruses to dinoflagellate ancestors, where they have eventually replaced histones as the primary chromatin packaging proteins [53,54,55].

Dinoflagellate nuclear DNA has a relatively high guanine and cytosine (G+C) content varying from 45%–70% [52,56,57]. It also hosts several modified nucleotide building blocks such as 5-methyl cytosine, N6-methyl adenine, and 5-hydroxymethyluracil (5-meU) that make it extensively methylated [58,59]. It has been suggested that 5-meU, that substitutes around 70% of the thymine (T), is associated with a restriction-modification system for discriminating between dinoflagellate and foreign DNA sequences thereby promoting stability in the open reading frames of the dinoflagellate genes [49,58,59].

Dinoflagellate nuclear genomes have acquired plastid targeted genes via successive horizontal gene transfer from the peridinin plastid, the tertiary replacement plastid and its host nucleus, cyanobacteria, red algae, haptophyte, and even bacteria, due to which the genomes are highly chimeric [60,61,62,63]. Despite this, their gene content is lower than expected, varying from 37–87 × 10^3^ protein coding genes, which accounts for only 0.05%–1.8% of the total genomic DNA [63]. However, this still remains larger than most other eukaryotes [63,64]. Two partial genomes from species belonging to the *Symbiodiniaceae* family, i.e., *Fugacium kawaguti* and *Breviolum minutum*, have been sequenced [56,65,66]. Recently the *Amoebophrya ceratii* (Syndiniales) genome was sequenced, which was substantially smaller than the *Symbiodiniaceae* genomes reported so far [67]. These studies show that dinoflagellates have uni-directionally aligned genes, forming cluster-like arrangements and have gene models that can be grouped into families [56,65]. Many of these genes are organized in multiple copies as tandem repeats, some of which may be present in up to ~10^5^ copies [68,69]. Despite the high gene copy numbers, the dinoflagellate genomes are mostly repetitive non-coding DNA. However, some dinoflagellate genes may have a low intron density and occasionally even lack introns, and these may be genes that are highly expressed [68].

Several ‘intron-less’ genes have resulted from the incorporation of cDNA back into the dinoflagellate genome through a process of *trans*-regulatory elements, or retroposition [70,71]. In the dinoflagellate genomes sequenced, >20% of genes appeared to be the result of retroposition [56,65,67,72]. Such *trans*-regulatory processes are mRNA processing reactions by which exons from two separately transcribed pre-mRNAs are joined [73,74]. This process also adds a 22 bp sequence, known as the spliced leader (SL), at the 5’ end of transcribed mRNA, possibly making the mRNA pool for translation [73,74]. SL *trans*-splicing acts to convert polycistronic pre-mRNA to monocistronic mRNA and also acts as a gene expression regulator [73,74,75]. However, SL *trans*-splicing in dinoflagellates is different compared to that in other organisms since the conserved binding motifs occur in the exons instead of the introns [73,75]. Dinoflagellate genomes appear to lack the *cis*-regulatory elements such as the TATA box, but have appeared to display TTTT(G) motifs that might have replaced the TATA box [65]. As a result of such factors, most genes appear to be post-transcriptionally regulated, with only 5%–30% being regulated at the transcriptional level [6].

The organelle genomes in dinoflagellates (both plastid and mitochondrial) are also remarkably different compared to other eukaryotes [63,76,77,78]. Most eukaryotic plastids contain circular genomes encoding 100–200 genes. However, peridinin containing dinoflagellates have highly reduced plastid genomes containing only 3–4 proteins that are encoded into plasmid-like ‘minicircles’ [78,79]. Similar to their nuclear genome, dinoflagellate organelle genomes have several gene copies that are frequently disrupted by fragments of other genes, and many of these genes are transferred to the nucleus [80]. Therefore, the peridinin-containing dinoflagellates encode the smallest number of plastid genes for any photosynthetic eukaryote [78]. The mitochondrial genomes in dinoflagellates are also highly duplicated and recombined, similar to the nuclear genomes, and are comprised of abundant non-coding and repetitive sequences that are rich in AT content, yet these genomes are highly reduced compared to other closely related protists, due to gene transfers to the nucleus [63,67,77].

The complex genetic machinery of dinoflagellates, as briefly described above, makes it tedious to verify certain genetic pathways in dinoflagellates [6,63,81]. Furthermore, dinoflagellates have associated bacteria that cannot be eliminated from cultures, which makes it difficult to fully exclude the impact of these associated assemblages on biosynthetic pathway studies [6]. With the aid of sequencing cDNA libraries to generate expressed sequence tags (ESTs), it became possible to study gene expression and regulation in dinoflagellates for which little-to-no genomic information is available [63]. ESTs serve as markers for genes expressed under specific conditions and can be used as probes in the recovery of full-length cDNA or genomic sequences, recognition of exon and intron boundaries, delineation of protein families, and development of probes for genome wide expression profiling [52,63,82,83]. Furthermore, transcriptomic surveys by way of next-generation sequencing (NGS) RNA-Seq, in particular, have illuminated our understanding of the unique biology, metabolism, and ecology of dinoflagellates [82,84,85]. In this review, we summarize our current understanding of the genetic basis of toxin biosynthesis in dinoflagellates and discuss the future directions for this field.

## 3. Dinoflagellate Toxins

Dinoflagellate toxins are structurally and functionally diverse and possess unique biological activities, including ion channel modulation, phosphatase inhibition, hemolysis, mycotoxicity, and cytotoxicity [86,87]. In organisms including humans, the voltage-gated ion channels, such as the sodium, calcium, and potassium channels are electric signal generators that control muscle contractions, hormone secretion, sensing of the environment, information processing in the brain, and the reflex output to the peripheral tissues/muscles [38]. These channel pores can get blocked by toxin molecules, preventing ion conductance and altering voltage dependent gating [38]. Traditionally, the biosynthetic pathways for these complex compounds have been elucidated by isotope labelled studies on the cultured microorganisms [40,88]. While these studies have not been able to comprehensively elucidate the entire biosynthetic pathways involved, they have provided us great insights into the structural complexity of these compounds [87,88].

### 3.1. Dinoflagellate Toxins: Polyketides

Polyketides are a highly diverse group of natural products with large and complex carbon structures that are assembled from simple acyl building blocks [89,90]. Most toxins currently linked to NSP, CFP, and DSP, as well as AZAs, PLTXs, YTXs, and cyclic imines, have a polyketide backbone and are broadly classified into three main categories according to their chemical structures; (a) polycyclic polyketides (e.g. BTXs, CTXs, YTXs, PLTXs, and maitotoxins), (b) macrolides (e.g., amphidinolides, pectenotoxins, and spirolides), and (c) linear polyketides (e.g., DTXs and OA) [87,88,91]. Much of our current knowledge on polyketide biosynthesis is obtained from bacterial and fungal products and their biosynthetic pathways, which have enormous commercial value and remain among the most successful candidates among natural drug discovery [91,92]. The enzymes responsible for polyketide biosynthesis are large multi-domain enzyme complexes known as polyketide synthases (PKSs) that resemble fatty acid synthases (FASs) in both structure and function [91,93,94,95,96,97,98]. PKSs have unusual structures and catalytic reactions that are essential for understanding enzymatic catalysis and protein–protein interactions among fundamental biological processes in dinoflagellates. Such knowledge will improve our knowledge of their ecological and evolutionary significance, and will also aid in harnessing their biological potential for medical and other biotechnological advancements [92,99].

#### 3.1.1. Polyketide Biosynthesis

**Figure 2:**
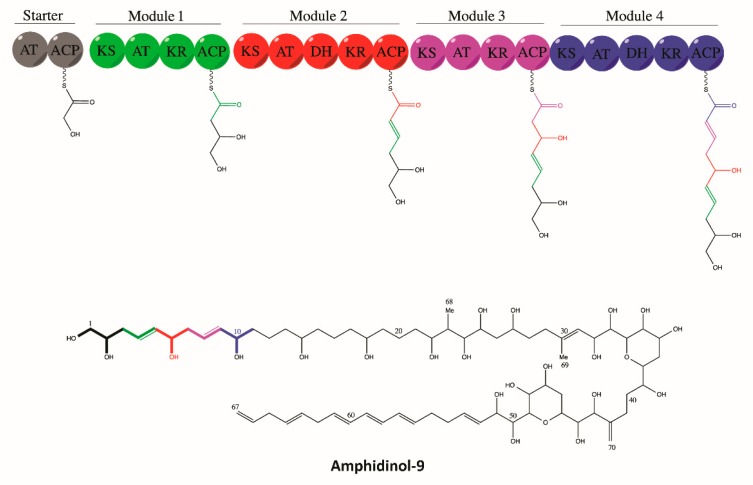
Polyketide biosynthesis. Schematic representing the putative biosynthesis pathway of polyketide compound Amphidinol-9 by various modular PKS domains represented by ACP: acyl carrier protein, AT: acyl transferase, DH: dehydratase, KR: ketoreductase, and KS: ketosynthase. Figure credit: Gurjeet Singh Kohli.

Polyketide biosynthesis is a product of sequential condensation reactions of small carboxylic acid subunits into a growing acyl chain, similar to fatty acid biosynthesis, performed by β-ketosynthase (KS) domains [91,94,97,100]. The growing carbon chain resides on an acyl carrier protein (ACP) that presents it to the catalytic sites of the KS domains, while an acyl transferase (AT) presents the extender units to be added to the growing chain [91,100]. These carbon chains can undergo optional reduction of beta-ketone to an alcohol, dehydration of the alcohol, and saturation of the resultant double bond through the activities of ketoreductase (KR), dehydratase (DH), and enoyl-reductase (ER) domains, respectively [89,100]. In addition, non-ribosomal peptide synthases (NRPSs) are also linked to the biosynthesis of polyketide molecules, especially the ones that contain an amide or amine group in their structure [101,102]. These large multi-modular domains catalyze the incorporation of amino acids into the polyketide backbone in a manner analogous to PKSs [102]. A typical NRPS module comprises of an initiation module, i.e., an adenylation domain (A) that specifically activates an amino acid, a peptidyl carrier protein (PCP), and a condensation domain (C) that is responsible for chain elongation by creating a peptide bond between two PCP-bound amino acids [101,102]. Finally, a thioesterase domain (TE) releases the full-length molecule from the PKS complex [91,100,103] (Figure 2).

PKSs are generally classified into three groups according to structural and functional elements (domain organization) [94,98]. Type I PKSs are large proteins comprising a set of catalytic domains on a single protein that are used in a repetitive fashion for chain elongation, similar to fatty acid synthesis in animals and fungi [94,98]. Type II PKSs comprise different multi-protein complexes, where each catalytic domain is on a separate peptide domain which function as mono-functional proteins in an iterative fashion analogous to type II FASs in bacteria and plants [94,98]. Type III PKSs are self-contained homodimeric enzymes, where each monomer performs a specific function in an iterative manner without the use of ACP proteins and acts directly on the acyl unit precursor molecules [91,98,100]. These PKSs are typically associated with chalcone and stillbene synthases in higher plants, but have also been identified in several bacteria and are similar to Type II PKSs, but smaller in size [91,98,100]. Similarly, FASs are classified as type I multi-domain proteins synthesizing lipids in the cytosol [104], or as Type II FAS complexes that catalyse lipid biosynthesis in the chloroplast stroma of plants [105].

PKSs and FASs are well documented amongst bacterial and fungal secondary metabolites, and studies over the past 15 years have also begun to report them in toxic dinoflagellates [81]. The common evolutionary history of PKSs and FASs, and their possible bacterial origins have been the subject of immense discussion [81,94,95]. Multiple studies over the last decade examining their evolutionary history and functional diversity have been put forward to fully comprehend the complex toxin biosynthesis pathways in dinoflagellates.

##### 3.1.1.1. The Eukaryotic Origin of Polyketide Toxin Biosynthesis

The first PKSs in dinoflagellates were identified by Snyder et al. [101] that reported partial type I, but not type II PKS genes from non-axenic cultures of six different dinoflagellate species using reverse transcription (RT) PCR. These species included OA producing *Prorocentrum lima* and *P. hoffmanianum*, BTX producing *K. brevis* and amphidinolide producing *Amphidinium operculatum* [106]. Partial type I PKSs were also identified from *Symbiodinium* sp. and *Gymnodium catenatum,* which were not known as polyketide producers at the time, thereby highlighting the potential to identify novel polyketides from these species in the future. This study did not demonstrate a clear phylogenetic origin as the KS domains were interspersed with bacterial and fungal type I KS sequences, and sequences from *K. brevis* branched into the clade with PKS encoding genes from the apicomplexan parasite *Cryptosporidium parvum* [106,107]. However, as 16S rDNA genes were amplified from the dinoflagellate cultures, it could not be ruled out that these were bacterial genes [106]. Snyder et al. [108] followed this study with the discovery of two putative PKS genes from *K. brevis* that were localized within the cell using both fluorescence in situ hybridization (FISH) and PCR screening, but also found these genes in the associated bacteria, as well as in *Amphidinium* strains that are non-brevetoxin producers [63]. This study presented the first evidence of resident PKS genes in any dinoflagellate, but could not comprehensively conclude if they were potentially derived from the associated bacteria [108].

The discovery of the conserved spliced leader sequence at the 5’ end of mRNA and the poly-A tail at the 3’ end from over 100 contigs from several different dinoflagellate species aided in identifying full length PKS transcripts from *K. brevis* [73,74,109]. This was pivotal in investigating toxin biosynthesis genes from dinoflagellates as it provided the most compelling evidence of dinoflagellate origin of these domains, as the process of trans-splicing on polycistronic mRNA does not take place in bacteria [73,74]. Since then numerous studies have reported PKSs from dinoflagellates with a 5’ spliced leader sequence and poly A tails, which phylogenetically group within the protist clade, thereby confirming their eukaryotic origin [81].

##### 3.1.1.2. FAS vs PKS in Toxin Biosynthesis

The structural and functional similarities between FASs and PKSs have caused considerable debate over the biosynthesis of these molecules in dinoflagellates [77,89]. Early research on fatty acid biosynthesis genes in dinoflagellates and other closely related organisms identified cytosolic type I FASs from the dinoflagellate *Crypthecodinium cohnii* [110], in contrast to type II FASs that were reported from the apicomplexans *Toxoplasma gondii* and *Plasmodium falciparum* [111]. These findings gave rise to the hypothesis suggesting multi-functional Type I FAS/PKS genes could have evolved from individual Type II FAS genes through one or more gene fusion events. Van Dolah et al. [112] used ^3^H acetate labelling of fatty acids in *K. brevis* and reported cytosolic incorporation of these molecules, with little incorporation in the chloroplasts. Such feature is typical of type I FAS systems [112]. Along with the absence of Type II FAS transcripts from the transcriptomic assemblies of *K. brevis*, these findings could only be supported by the presence of a Type I FAS system in *K. brevis*. However, no sequences with Type I FAS conserved domains could be reported from the transcriptome, which raised the question of whether dinoflagellate fatty acid synthesis was carried out by PKSs [112]. Pawlowiez et al. [113] constructed a cDNA library from the CTX -producing *G. polynesiensis* and identified 33 PKS related sequences, but none of the KS domains reported in this study were mapped to the fatty acid biosynthesis pathway, which provided further evidence for fatty acid biosynthesis possibly being carried out by PKSs [113].

Kohli et al. [81] mined the transcriptomic libraries of 24 genera and 46 strains of dinoflagellates that were sequenced under the Marine Microbial Eukaryote Transcriptome Sequencing Project (MMETSP) and screened them for the seven key enzymes involved in fatty acid biosynthesis, i.e. 3-ketoacyl ACP synthase I, II and III, ACP S-malonyl-transacylase, trans-3-ketoacyl ACP reductase, 3-hydroxyacyl-ACP dehydratase and enoyl-ACP reductase. This study confirmed the eukaryotic origin of FASs as well as their type II like structure, by identifying 5’ spliced leaders and poly A tails on them as well as detecting transit peptides targeted towards the chloroplast [81]. The authors postulated that these type II FAS genes have transferred from the cytosolic plastids over to the nucleus as they are under a strong selection pressure owing to them coding for essential life processes [81]. This study clearly separated FASs from PKSs and thus provided a good framework to investigate the different functionality of these genes and the biosynthetic pathways in dinoflagellates [81].

##### 3.1.1.3. KS Domains: Phylogenetic and Structural Diversity

Most studies investigating PKS toxin biosynthesis have targeted the KS domains as they are the most conserved among the PKS genes [114]. These domains have the greatest potential for revealing divergent homologs and thus provide the most information on the evolutionary history of PKS genes in dinoflagellates, and protists in general [114,115]. Early phylogenetic analysis performed on protists by John et al. [115] using the genomic data from Chromalveolate, Excavate and Plantae species, along with other known protist and bacterial PKS sequences, showed sequences with high similarity to type I PKS genes were present in only a few lineages. Besides the patchy distribution of type I PKSs, a new modular type I PKS gene cluster from protists was identified that differed from previously known type I PKS clusters [115]. Following studies on both toxic and non-toxic strains of *K. brevis* grouped the dinoflagellate derived KS contigs within the aforementioned type I PKS clade containing sequences from other protists [109,116]. While the *K. brevis* sequences clustered with type I PKSs, their structure and size were more similar to type II PKSs, with the presence of individual catalytic domains on separate transcripts [109,116]. Since that time, single domain PKSs have been reported in numerous toxic and non-toxic dinoflagellate strains/species [113,117,118,119,120,121,122,123]. Eichholz et al. [117] reported two dinoflagellate specific clades within the ‘protistan’ clade, one that was comprised of sequences from all three dinoflagellate with typical KS amino acid motifs and the other clade containing only sequences from *K. brevis* with alterations in their active sites [117].

**Figure 3:**
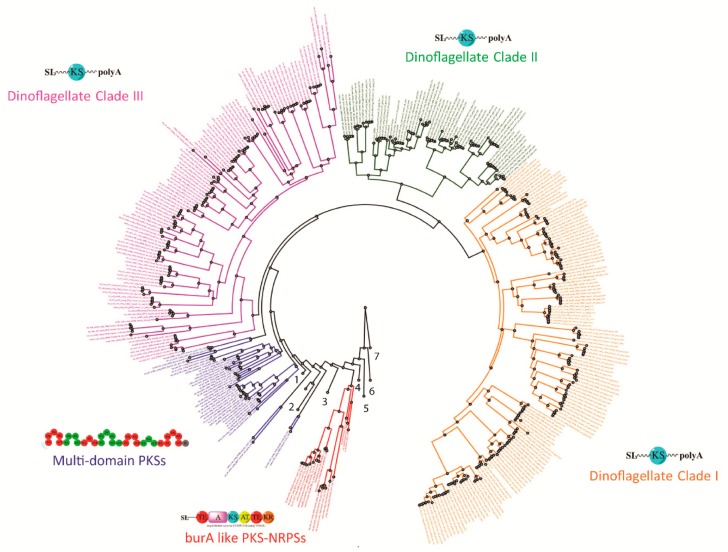
Ketoacyl synthase (KS) domain phylogeny in dinoflagellates (modified from [81,122,129]). Phylogenetic tree representing the three sub-clades of KS domains within the dinoflagellate type I PKSs, multi-domain PKSs that cluster with other apicomplexans (condensed clade represented by 1), chlorophytes, haptophytes (condensed clade represented by 2) and the *bur*A like PKS-NRPSs reported from dinoflagellates along with schematic representations of PKS domains represented by AT: acyl transferase, A: Non-Ribosomal Peptide Synthase, KS: ketosynthase, DH: dehydratase, KR: ketoreductase, TE: thioesterase, ACP: acyl carrier protein, ER: enoyl reductase, SL: 5’ splice leader, polyA: poly A tail. Other PKS groups are represented by 3: Modular type PKS *cis* AT clade, 4: fungal PKS non-reducing clade, 5: Animal type I FAS, 6: fugal PKS reducing; and 7: Type II PKSs.

**Figure 4:**
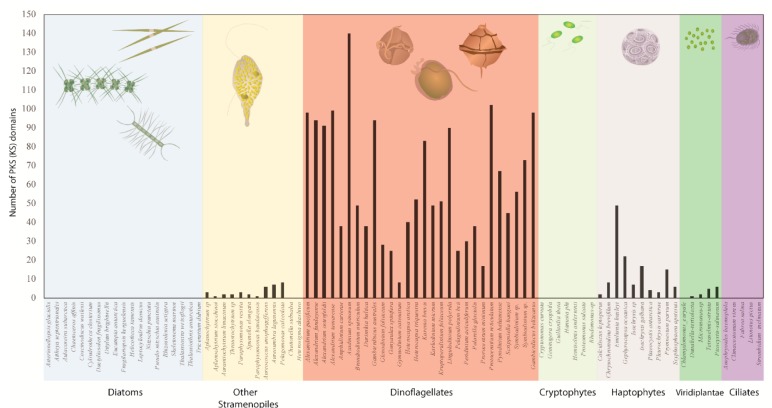
Survey of ketosynthase (KS) domains from various marine microbial eukaryotic lineages (modified from [81]). The figure represents the large number of KS domains that have been reported from the analysis of MMETSP libraries of different dinoflagellate species in comparison with species of cryptophytes, haptophytes, viriplantae, ciliates, diatoms, and other strameopiles. Photo credit: Diana Kleine, Jane Thomas and Tracey Saxby, Integration and Application Network, University of Maryland Center for Environmental Science (ian.umces.edu/imagelibrary/).

With more comprehensive transcriptomic sequencing and genome wide surveys of several dinoflagellate species, numerous sub-clades containing both mono- and multi-functional domains have now been reported (Figure 3). These domains closely match with sequences from other dinoflagellates as well as species of apicomplexans, chlorophytes, and haptophytes among the type I ‘protistan’ clade. Such phylogenetic analysis suggest that these genes might be involved in the production of polyketide compounds that are produced by all species since no clear pattern based on chemical structure of the toxic compounds could be established [81,119,120,122,124,125]. Dinoflagellates have a much larger number of unique KS domains compared to other protists, and possibly all other eukaryotes [81] (Figure 4). Kohli et al. [81] extracted a total of 2577 KS domains from 24 genera comprising 46 strains of dinoflagellates, which were broadly clustered into three distinct type I single domain KS sub-clades within the dinoflagellate type I PKSs (Figure 3). Each of these clades included sequences from numerous species that are clearly not related to dinoflagellate species-based phylogeny suggestive of frequent, and possibly multiple, gene duplication events, horizontal gene transfer (HGT), domain shuffling and losses, and recombination have occurred in their genomes [81,125]. The conserved active site amongst KS domains (Cys-His-His), which is essential for their functionality, was observed in almost two-thirds of the domains, thereby suggesting novel functionality of these domains among dinoflagellate species [81].

Eichholz et al. [117] identified a previously uncharacterized conserved motif (ExExGYLG) at the N-terminal of the KS domains and speculated its function to be related to the mono-functional nature of KS domains and may play a role in structural rearrangements, substrate docking, or protein-protein interactions [117]. The conserved motif and its variants G(D/H/Y)YLG were observed at the 5’ end along with those containing another variant, G(A)LLG. These N terminal conserved sites have since been observed in other EST libraries [113,121,123]. The alteration of the conserved N terminal motif in the KS transcripts could explain the alternations/diversification in polyketide synthesis or the possibility that these copies might have diverged to play a role in other pathways via multiple gene duplication, loss, and selection events [113,117]. In contrast, no conserved domains were observed in the C-terminal region, thereby speculating that this region acts as a linker structure where the single domains could be separated to allow for the structural function of the complex enzymes [121].

##### 3.1.1.4. Other PKS Genes

The minimal domains required for the PKS condensations reactions are the ACP, KS, and AT domains [87,88,91,100,106]. However, the work of auxiliary PKS domains yields a great diversity of polyketide compounds from dinoflagellates [88,106]. Kubota et al. [126] used degenerate PCR primer sets designed especially for type I KS domains and identified KS domains from an amphidinolide producing *Amphidinium* strain. In the same study, using shotgun sequencing, gene products showing similarity to AT, DH, KR, ACP, and TE were also identified. This study also highlighted the presence of introns among PKSs that caused difficulties in cloning the entire genes from genomic DNA library [126]. Bachvaroff and Place [68] also identified KS and KR domains, this time from the EST library of *A. carterae*, and reported up to 18 introns from the KR sequences that were surveyed. This suggests that PKS domains are intron-rich which was further confirmed with the genomic surveys of *Symbiodiniaceae* species [68,124,125]. All polyketide biosynthetic enzymes needed to synthesize the complex polyketide backbones, i.e. KS, KR, AT, ACP, ACP synthase (ACPS), TE, and ER, have been identified in several toxic dinoflagellates [119,120,121,123]. However, despite several transcriptomic and genomic surveys, only a handful of these domains have been investigated further for the better understanding of their evolutionary function and purpose in toxin biosynthesis.

###### Multi Domain PKSs

Deep sequencing of dinoflagellate transcriptomic libraries have yielded contigs encoding multiple PKS domains per transcript [113,122]. Initially, the dinoflagellate-specific spliced leader was not identified on these transcripts, and their clustering with bacterial KS domains suggested that these contigs were most likely part of the bacterial contamination within the dinoflagellate culture, since the cultures were not axenic [120,122]. Bachvaroff et al. [127] identified a hybrid PKS-NRPS transcript from *A. carterae* that encoded the dinoflagellate-specific spliced leader sequence and poly A tail that clustered with bacterial type I PKSs (Figure 3). Such domain architecture is reminiscent of the *bur*A gene in bacteria *Burkholderia* that are PKS-NRPS hybrids and are involved in the synthesis of polyketide precursors in burkholderic acid biosynthesis [128]. These PKS-NRPS hybrids are potentially widespread among dinoflagellates as they have been identified from species that produce amine/amide-containing compounds, such as *K. brevis* and *Ostreopsis* spp., and also from species that do not produce any known products that would involve the NRPS pathway, e.g., *Gambierdiscus* spp. [122,123,129]. Such reports suggest that these domains were acquired prior to the diversification of dinoflagellates through horizontal gene transfer from cyanobacterial genes [118,122,123,124,130]. Dinoflagellate genomes are also subject to frequent recombination events and gene duplications, which play an important role in the evolution of modularity in PKSs. There is increasing evidence of multifunctional PKS domains in several dinoflagellates, indicating that multi-functionality coevolves with mono-functional domains [125]. However, it is still unclear whether the single domains PKSs have evolved from the decomposition of Type I multifunctional PKS clusters, or fusion of mono-functional PKS domains has led to multi-functionality [125].

Recent genomic surveys of PKS-NRPS domains in *Symbiodiniaceae* species demonstrated a phylogenetic trend specific to amino acid substrate utilization (amongst A- domains) and functional categories (in C-domains), as opposed to a species phylogeny [125]. This demonstrates that the chemical diversity in amine/amide-containing polyketide molecules is driven by the incorporated NRPS domain [125]. In such hybrids, a sequence of amino acids within the A-domain catalytic pocket governs the recognition and activation of amino acid substrates [125]. Therefore, any point mutations within the catalytic domain can drastically change the specificity of the A-domain. Single A- domains tend to incorporate polar and non-polar amino acids and interact with other NRPS components by transferring the substrate to the C- domain, which can perform epimerization or cyclization of the amino containing polyketide molecule [125].

###### Acyltransferase (AT) Domains

The acyltransferase (AT) domains recognize and attach carboxylic acid building blocks onto an ACP domain, which are then incorporated into the polyketide backbone by the catalytic reactions of the KS domains [89,96,100]. AT domains are broadly classified based upon specificity towards the building units [89,96,100,131]. *Cis*-AT PKSs are specific for methyl malonyl-CoA, hydroxyl malonyl-ACP, and methoxy malonyl-ACP, whereas *trans*-AT PKSs are more specific for malonyl-CoA only [131,132]. AT domains have been reported from several dinoflagellate species but have not been investigated in detail [120,121,123,125,126]. Recent genomic surveys of *Symbiodiniaceae* species by Beedessee et al. [125] reported a large number of *trans*-AT domains from these species and demonstrated a clear demarcation between *cis*- and *trans*- AT domains [125]. *Trans* ATs were first identified in bacteria and have since been reported from several microalgal species [131,133]. They are known to have unique catalytic mechanisms, highly unusual architectures with modules carrying either novel catalytic domains, or domain orders [132,134]. These domains have also been known to recombine via gene duplication, domain shuffling and retroposition, to form novel gene clusters in a mosaic like fashion, thereby promoting diversification of PKS biosynthesis. [125,134]. On the other hand, it is hypothesized that *cis*- AT domains may have been acquired via horizontal gene transfer of the entire PKS module, but remains to be investigated [125].

###### Ketoreductase (KR) Domains

Ketoreductase (KR) domains have been reported from several dinoflagellates to date, however the number of unique single KR domains identified from dinoflagellates are much smaller compared to the number of KS sequences in those species suggesting that the selection of KR domains is more conserved compared to the KS domains [121,122,123,129]. Recent phylogenetic investigations on KR domains placed the dinoflagellate derived KR domains into one large single domain protist clade, similar to KS domains, along with five distinct multi-KR domains sub-clades [123,129]. Sub-clade 1 comprised of KR domains from multi PKS modules with ‘KS-KR-ACP’ domain architecture, whereas sub-clade 2 contains KR domains from *trans*-AT PKS modules with ‘KS-DH-KR’ domains. Sub-clade 3 KR domains possess an ER domain inserted between the N-terminal and C-terminal, which has previously been described from FAS-like domains. Subclades 4 included all PKS-NRPS hybrids similar to *bur*A-like sequences from dinoflagellates and *Burkholderia* spp. [123,129]. Van Dolah et al. [123] reported sub-clade 5 to include diverse multi PKS contigs with ‘KS-DH-KR’ architecture, along with PKS-NRPS hybrids and a multi-module PKS with highly amplified ACP domains and potential MT domains at their C-terminal ends [123]. Such phylogenetic analysis reveals that KR domains cluster according to their module architecture rather than grouping based on species or the toxic compound produced.

Functionally, KR domains have been classified based on the stereochemistry of the products they catalyze; i.e., A-type KRs produce an *S*-hydroxyl group and B-type KRs that produce the *R*-hydroxyl group [135]. An A-type KR would contain a conserved tryptophan residue (W motif) and generate an L-3-hydroxyacyl intermediate, whereas B-type KRs contain a conserved Leu-Asp-Asp (LDD motif) [136]. Among toxin producing dinoflagellates, several stereoisomeric analogues have been reported which do not share the same biological activity compared to other analogues. Such structure-activity relationships in toxin analogues could be driven by different KR domains that might be giving the molecules their varying toxigenic properties [129].

###### Methyl Transferase (MT) Domains

Both cyclic and non-cyclic polyketides constitute repetitive methyl groups that are derived from *S*-adenosyl methionine (SAM) [88,137,138]. MT domains catalyze the transfer of methyl group from SAM to either the carbon, nitrogen or oxygen atoms at various positions on the PKS-NRPS backbone and have been classified as C-MT, N-MT, and O-MT (carbon, nitrogen, and oxygen, respectively) depending upon their site of methylation [139,140]. MT domains have not been well documented in dinoflagellates. Only recent studies have unraveled multi-domain PKSs comprising KR-MT domains from several toxic and non-toxic dinoflagellate species [120,123,129]. These domains are known to act as linker regions that adopt a short chain reductase fold in the structure of large polyketides and might play a vital role in introducing folds in the structure of large toxin molecules such as PLTX and MTX and their analogues [129,141,142,143,144].

##### 3.1.1.5. Other Genes

Through systematically assessing the functional capacity of 47 transcriptomic libraries from eight different dinoflagellate orders, Stephens et al. [145] identified a significant overrepresentation of membrane transporter gene families in toxin producing dinoflagellates, particularly the Voltage-gated Ion Channel (VIC) superfamily and the monovalent Cation: Proton Antiporter-1 (CPA1) family that are critical in the maintenance of ion concentrations and gradients across cell membranes. Even though these genes are not directly involved in toxin biosynthesis, they facilitate the secretion of toxins and make the producing cells resistant to their own toxins [145]. Most dinoflagellate toxins are known to target the sodium/calcium voltage-gated ion channels [38]. In eukaryotes, these channels are highly glycosylated with sialic acid, which is known to modulate the excitability of voltage-gated ion channels [146]. Stephens et al. [145] reported an overrepresentation of glycosyltransferase family and sialyl-transferase activity in toxic dinoflagellates. This indicates that the voltage-gated ion channels in toxin producing dinoflagellates are resistant to their own toxins due to processing and attachment of sialic acids to other macromolecules [145]. However, the use of sialic acid to either increase or recover the activity of toxin-resistant channels in dinoflagellates remain to be fully understood.

##### 3.1.1.6. PKS Gene Regulation

Gene regulation and its role in toxin biosynthesis still remains unclear in dinoflagellates since the expression levels of most transcripts in toxic dinoflagellates remain unchanged despite varying culturing conditions or growth phases [147,148,149,150]. Transcription factors are less abundant among dinoflagellates in comparison to other protists, whereas post-transcriptional regulation remains the more dominant regulatory feature of gene expression in these organisms (for a review, see Roy et al. [151]). Most, if not all, of the transcripts in dinoflagellates are post-transcriptionally modified at their 5’ end by *trans*-splicing, which converts polycistronic pre-mRNAs into mature single gene messages [73,74]. The SL *trans*-splicing mechanism was first identified in trypanosomes, which carry out continuous transcription and rely entirely on post-transcriptional mechanisms for gene expression [152]. The SL *trans*-splicing mechanism has now been identified in numerous dinoflagellate species [73,74], and may be an essential requirement for various processes in dinoflagellates that are post-transcriptionally regulated such as bioluminescence [153], carbon fixation [154], photosynthesis [155], circadian regulated processes [147,150], acute stress responses [156], and responses to nitrogen or phosphorus limitation [157].

Microarray analysis in *K. brevis* showed the expression of PKSs did not change over the diel cycle despite evidence that toxin production is specific to certain diel phases or cell cycle stages in dinoflagellates [147]. Monroe et al. [116] compared the KS domain transcript levels between toxic vs. non-toxic strains of *K. brevis* and did not find any significant variation. However, at the protein level, KS domain proteins were 55%–70% less expressed in the non-toxic strain, suggesting that the ‘non-toxic’ phenotype in dinoflagellates might not be the result of gene mutations causing a non-functional PKS machinery, but rather a consequence of altered expression levels and/or activity of intact PKS protein complexes [116].

A number of studies have started to report a rich diversity of microRNA (miRNA) in dinoflagellates using a transcriptomic approach [65,158,159,160,161,162]. Additionally, small (or short) interfering RNA (siRNA) have been linked to the regulation of a large fraction of protein coding genes and processes [159]. EST libraries of *Alexandrium tamarense* have also reported miRNAs that are targeted towards housekeeping genes such as crystalline ribonucleoprotein complex and ribosomal proteins [160]. Genomic surveys of *Symbiodiniaceae* species have yielded numerous genes involved in primary metabolism of cells that are controlled by miRNAs [65,159]. However, the regulation of PKS toxin biosynthesis by miRNA in dinoflagellates remains to be established.

### 3.2. Dinoflagellate Toxins: Cyclic Imines

Cyclic imine (CI) toxins are a class of fast-acting neurotoxins that include spirolides, gymnodimines, pinnatoxins, as well as other minor compounds produced by a number dinoflagellate species: *Alexandrium ostenfeldii*, *Karenia selliformis*, *K. mikimotoi*, *Prorocentrum lima*, *P. maculosum,* and *Vulcanodinium rugorum* (for a review, see Stivala et al. [163]). Most of them are yet to undergo extensive transcriptomic studies to identify PKS or other toxin-related genes. A biosynthetic pathway for spirolide C has been proposed on the basis of results from studies using radiolabeled precursor molecules [164,165], and a hypothetical mechanism for pinnatoxin production has also been presented [166]. The structural similarities of spirolides, gymnodimines and pinnatoxins suggest at least a partially shared genetic basis, and it has been hypothesized that spirolides may represent the results of a horizontal gene transfer and recombination event involving gymnodimine and pinnatoxin genes [167]. The few transcriptomic studies have revealed the presence of several PKS genes in strains of spirolide-producing *A. ostenfeldii* [69,117]. However, the detailed genetic background of CI toxin production remains unknown and specific PKS genes have not yet been directly linked with SPX, GYM, or any of the other toxic CI natural products.

### 3.3. Dinoflagellate Toxins: Alkaloids

Paralytic shellfish toxins (PSTs) are alkaloid neurotoxins produced by dinoflagellates as well as by prokaryotic cyanobacteria. Saxitoxin (STX) is the parent PST compound originally purified from the clam *Saxidomus giganteus* [168]. PSTs have a tricyclic perhydropurine backbone, and structural variation occurs due to differing functional groups, resulting in compounds such as the C-toxins, the gonyautoxins (GTX), and the saxitoxin and neosaxitoxin group (e.g., STX and NEO) [169]. In vertebrates, PSTs prevent the transduction of neural signal by blocking voltage-gated ion (Na^+^, K^+^, Ca^2+^) channels, causing paralytic shellfish poisoning (PSP) [170,171,172,173]. Because of its high toxicity, STX has been enlisted as toxin relevant to bioterrorism [174].

**Figure 5:**
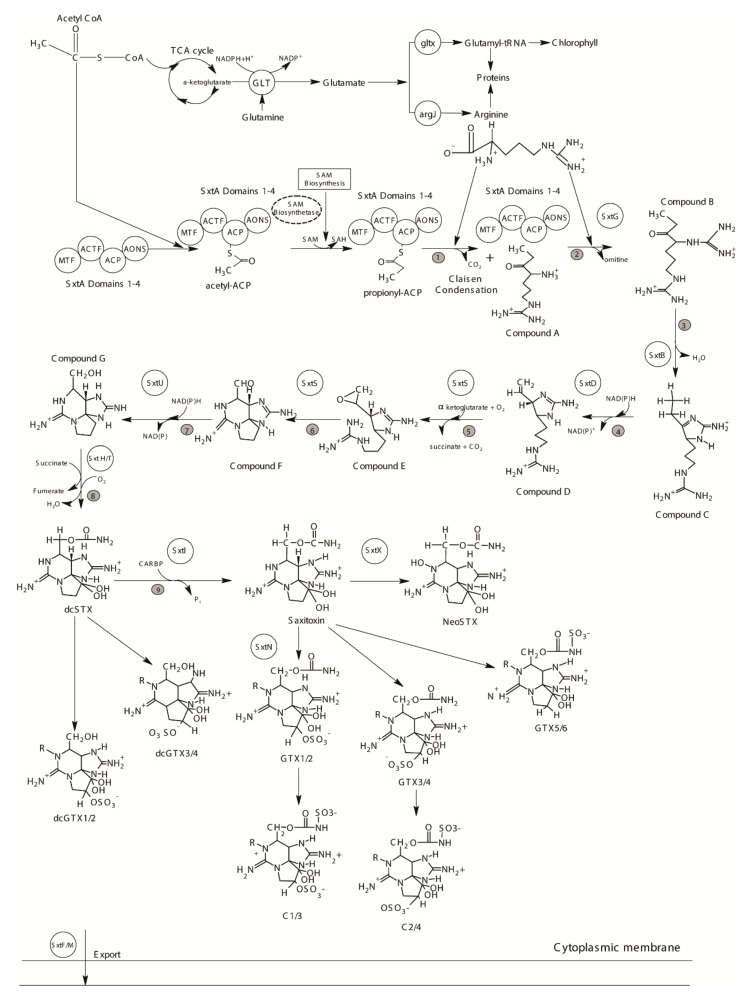
Revised biosynthetic pathway of PSTs in dinoflagellates (modified from [22,184,185,193,195]). The reaction steps are as follows; **1**: Claisen condensation; **2**: amidino transfer; **3**: heterocyclization; **4**: desaturation (Double bond formation); **5**: epoxidation of the new double bond; **6**: aldehyde formation; **7**: terminal aldehyde reduction; **8**: dihydroxylation and **9**: carbamoylation.

PSTs reported in the dinoflagellates can be classified as non-sulfated (STX, NEO), mono-sulfated (GTX 1-6), di-sulfated (C 1-4), decarbamoylated (dcSTX, dcNEO, dcGTX1-4), deoxy-decarbamoyated (doSTX, doGTX1-2) and GC toxin analogues (only reported in *Gymnodinium catenatum*) [38,171,175,176,177] (Figure 5). The addition of one or more sulfate groups in the basic STX skeleton decreases the toxicity of the resulting molecule compared to the non-sulfated variants [171]. For this reason, STX and NEO are considered to be the most potent PSTs, followed by the gonyautoxins and C- toxins, which contain either one or two sulfate groups, respectively [171]. Approximately eight species of the dinoflagellate genus *Alexandrium,* as well as the species *Gymnodinium catenatum* and *Pyrodinium bahamense var compressum,* are known to produce PSTs [178,179,180,181].

#### 3.3.1. *sxt* Genes and PST Biosynthesis Pathway in Dinoflagellates

The PST biosynthesis pathway has been studied extensively by investigating radioisotope labelled precursor incorporation since the 1980s [182], but the genes putatively involved in this process, i.e., the *sxt* gene cluster, were first described from cyanobacteria over 20 years later, and have since been reported from species of approx. seven cyanobacterial genera [148,183,184,185,186,187]. The prokaryotic cyanobacteria and eukaryotic dinoflagellates are only remotely related, but PSTs appear to be synthesized through a similar biosynthetic pathway using the same precursors in both groups of organisms [185,188] (Figure 5). Fourteen of the *sxt* genes (*sxtA* – *sxtI*, *sxtP* – *sxtS* and *sxtU*) have been identified as being common in PST-producing cyanobacteria and are considered “core genes” [185,189]. Amongst them, 8 *sxt* genes appear to be directly involved in the biosynthetic process [185] (see Figure 5).

**Figure 6:**
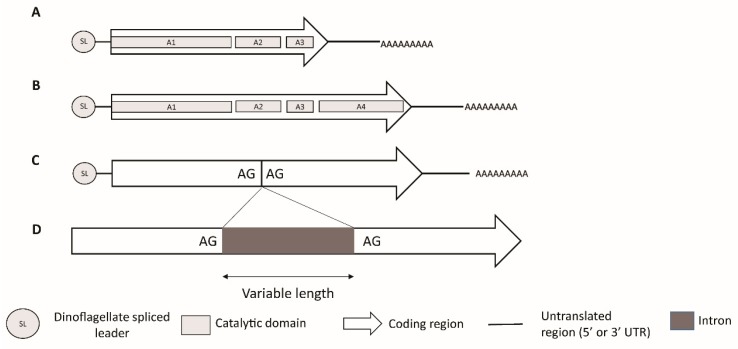
The structure of *sxt*A and *sxt*G in dinoflagellates (modified from [57,191]). **A.** Transcript structure of *sxt*A short isoform. **B.** Transcript structure of *sxt*A long isoform. **C.** Transcript structure of *sxt*G. **D.** Genomic structure of *sxt*G.

The identification of the *sxt* genes in cyanobacteria provided a platform for the discovery of corresponding genes in dinoflagellates [57,190,191,192]. Transcriptomic annotation with the help of cyanobacterial *sxt* gene sequences as well as PCR and amplicon sequencing technologies have led to the identification and characterization of *sxt* homologs in dinoflagellate species [57,190,191,192]. The discovered *sxt* sequences display features characteristic of dinoflagellate genes; i.e., they have a high (~65%) GC content and are expressed as monocistronic transcripts with a 5’ splice leader sequence and a 3’eukaryotic poly-A tail [122,191] (Figure 6).

In both cyanobacteria and dinoflagellates, STX biosynthesis is initiated by the enzyme *sxtA* [185,188] (Figure 5). It contains four catalytic domains, encoded by *sxtA1* (SAM-dependent methyltransferase, MT), *sxtA2* (GCN 5-related N-acetyltransferase, ACT), *sxtA3* (acyl carrier protein, ACP), and *sxtA4* (8-amino-7-oxononanoate synthase like class II aminotransferase, AONS) [185]. The biosynthesis process starts with the binding of an acetyl-CoA derived acetyl with the ACP of *sxtA* to form acetyl-ACP, which is converted to propionyl-ACP after methylation of the acetate [185]. The AONS domain catalyzes a Claisen condensation between arginine and propionyl-ACP, thus producing ‘Compound A’ [185] (Figure 5).

In dinoflagellates, at least two *sxtA* mRNA isoforms have been reported as being simultaneously expressed [57]. Rather than a single homolog, *sxtA* appears to represent a gene family, with multiple paralogous copies, but only one of these copies appears to be linked to species that synthesize STX [190]. Phylogenetic analysis of *sxtA1* reported three paralogs, or clades, of which two, i.e., clades 1 and 3, appear to be widely present in non-STX producing dinoflagellates indicating possible gene duplications events [190]. However, clade 2 of *sxtA1* domain appeared to be exclusively made up of STX producing species [190]. The *sxtA4* domain was found to be present and highly conserved in several strains of eight species of STX-producing dinoflagellates, but was absent from the non-producing species, thereby confirming the hypothesis that *sxtA4* is an essential domain for STX biosynthesis [57,185,189,190]. Zhang et al. [193] used differential expression analysis to report that some paralogs are unrelated to STX biosynthesis, and further confirmed that the *sxtA4* domain is essential for STX biosynthesis since no expression of the long *sxtA* transcript isoform (including the domain *sxtA4*, as represented in Figure 6) was found in a non-toxic strain of *Alexandrium pacificum* (Figure 6). However, these transcripts were strongly expressed in an STX-producing strain of the same species [193]. At the same time, no significant differential expression of the short *sxtA* transcript (excluding *sxtA4*, as represented in Figure 6) was detected between the two investigated strains [193]. Interestingly, neither *sxtA1* nor *sxtA4* were found in the recently discovered non-toxic dinoflagellate species *Centrodinium punctatum*, which appears to be closely related to *Alexandrium* spp. [194].

The second core gene in dinoflagellate STX biosynthesis is the *sxtG,* which has been characterized at both transcript and genomic levels (Figure 6), and encodes for an amidinotransferase [57,185,191]. *sxtG* catalyzes the transfer of a guanidine group from a second arginine to the growing SXT backbone forming Compound B [185] (Figure 5). Like *sxtA*, dinoflagellate *sxtG* is homologous to its cyanobacterial counterpart, but has acquired eukaryotic features over the course of its evolution [191]. Though introns are generally not common in dinoflagellate genes, introns of variable lengths have been reported in *sxtG* in some *Alexandrium* spp. [191] (Figure 6). Initially, it was reported that *sxtG* was present in all *Alexandrium* spp. independent of STX production [57,191,196]. However, using qPCR, *sxtG* could not be amplified in the STX-producing *Alexandrium* species [181]. Additionally, no *sxtG* homologs were found in non-STX producing dinoflagellates in a study examining transcriptomes [190]. The result suggests that if *sxtG* is present in non-STX producing species, it may be transcribed at extremely low levels [191].

Apart from *sxtA* and *sxtG,* other toxin-related genes have not been characterized at the genomic level, and are known from transcriptomic studies involving several PST-producing dinoflagellate species [57,190,191,192,193,195] (Table 1). These include the remaining core *sxt* genes, which catalyze reactions starting from the third step of the PST biosynthesis pathway, a heterocyclization reaction by *sxtB* [185] (Figure 5). The following double bond formation is catalyzed by *sxtD*, and the subsequent synthesis of the second and third heterocycles via epoxidation and aldehyde formation by *sxtS* [184,185] (Figure 5). Following these steps, the enzymes *sxtU* and *sxtH/T* work in sequence to produce dcSTX, the first complete PST analogue, while one further step, carbamoylation by *sxtI*, produces the parent compound STX [184,185] (Figure 5).

#### 3.3.2. Concepts on *sxt* Gene Regulation

Complete understanding of toxin biosynthesis in dinoflagellates is challenging due to the unusual genomic features and gene regulation systems in dinoflagellates, as reviewed above. Large percentages of dinoflagellate DNA consists of non-coding repeat sequences with multiple tandem repeats of protein coding genes [64,198,199,200]. Mechanisms such as partial gene duplication and retroposition contribute to this copy number increase, and environmental stress may promote it [71,72,201]. An estimated 5%–30% of the dinoflagellate genes appear to be regulated at the transcriptional level, whereas most other genes may be regulated at the post-transcriptional or translational levels [6,202]. Dinoflagellates can take advantage of the abundant gene copies to help them produce more transcripts [200]. This mechanism is commonly called the ‘dosage effect’, and has also been reported from several other eukaryote organisms [75,203,204]. A study on *A. minutum* has suggested positive correlation between the genomic copy numbers of *sxtA4* with a total cellular PST content [205].

The possibility of transcriptional control of PST biosynthesis has been investigated in *Alexandrium* spp., and the hypothesis has been supported by the discovery of putative *sxtZ* homologs, a gene which in cyanobacteria is thought to be involved in the transcriptional regulation of STX biosynthesis [193], as well as the significant positive correlation between *sxtA4* expression levels and cellular PST quotas (Q_t_) under nutritional stress [206]. Whereas a less clear-cut correlation between the expression levels of *sxtG* and putative *sxtI* and Q_t_ were reported, an evident effect of macronutrient availability on *sxt* gene expression was observed [206]. In contrast to these findings, other studies have reported no correlation between *sxtA1* and *sxtG* mRNA quantities and toxin contents in strains of *A. minutum* [207], and no significant variation in expression levels of the toxin-related gene *sxtA4* in *A. pacificum* or genes *sxtA*, *sxtB*, *sxtD*, *sxtF/M*, *sxtG*, *sxtH/T*, *sxtI*, *sxtO*, *sxtP*, *sxtU*, *sxtW*, *sxtX*, *sxtZ*, *sxtPER,* and *sxtACT* in *A. pacificum,* as observed between growth stages, while at the same time the intracellular toxin content varied significantly [195,208]. The presence of grazers, while increasing the toxin content and diversity of PST structural variants, did not increase the number of *sxtA* transcripts in *Alexandrium catenella* [209]. Together, these findings suggest that the PST biosynthesis genes in dinoflagellates are under a complex regulatory system that may involve genomic, transcriptional as well as post-transcriptional and translational elements.

Several proteomics studies have been performed to provide insight on the mechanisms and possible translational regulation of STX biosynthesis. Correlation between the PST biosynthesis pathway and other carbon and energy utilizing pathways has been observed [210,211]. Proteins involved in the translational machinery, photosynthetic pigment production, and toxin biosynthesis with linkages to arginine, which is an important PST precursor molecule, and glutamate biosynthesis, were found to be upregulated during the same stage of the cell cycle [211,212,213]. Proteins involved in bioluminescence have been observed to be upregulated during toxin production [211] and found to be downregulated in a non-toxic strain with toxin related proteins [210]. Hence, it can be concluded that the toxin biosynthesis might not be regulated as a single pathway rather than being regulated in concert with other biosynthetic pathways.

#### 3.3.3. Applications for Detection and Monitoring of PST-Producing Dinoflagellates

The advances in characterization of the dinoflagellate STX biosynthesis pathway have made it possible to study the role of gene copy numbers and *sxt* gene expression in the regulation of PST biosynthesis [205], as well as to concentrate efforts on the development *sxt*-gene based monitoring assays [214,215]. The unprecedented socio-economic impacts of blooms of PST-producing dinoflagellates have driven the efforts to mitigate their impacts [34]. One approach is to use molecular methods such as quantitative polymerase chain reaction (qPCR) to detect and monitor PST-producing species. Several qPCR assays for the detection of *Alexandrium* spp. have been developed based on rRNA genes (e.g., References [216,217,218,219,220,221]), with specificities and sensitivities down to one cell per litre [221]. However, these assays do not provide any indication regarding the potential toxicity of a bloom as they only detect the presence of the *Alexandrium* cells.

Furthermore, studies have shown that rRNA gene copy numbers are highly variable [216,217,222], and this may lead to over- or underestimation of cell densities [221,223]. Characterization of *sxt* genes has made the development of PST-producing strain specific qPCR assays [214,215,224,225], and studies have shown that the genomic copy numbers of *sxt* genes vary less compared to rRNA genes, allowing for more accurate cell density estimates [197,223,224]. These assays have been successfully trialed to indicate the presence of toxic strains in seawater [215,224,225,226], and in commercially harvested oysters [227]. The detection of the toxic strains at low concentrations in seawater, enabled by *sxt* gene-based qPCR assays, provides a means for early warning systems designed to detect developing harmful blooms. In the future, combining qPCR with other analysis methods such as meta-transcriptomics could provide even more information on the active metabolic processes related to toxin production throughout bloom events [228].

## 4. Concluding Remarks

Immense advances have been made in the study of PST biosynthesis and in its application to the aquaculture industry. However, the studies on PKS domains and their relationships to polyketide-based toxins and cyclic imines have barely begun. Most toxin producing dinoflagellates are yet to undergo extensive transcriptomic studies to identify PKSs or other toxin-related genes. PKSs reported from the transcriptomic and genomic surveys of dinoflagellates have yielded divergent homologs, belonging to different phylogenetic sub-clades and producing a diverse range of secondary metabolites, however no toxin or species-specific sub-clades have been identified. Additionally, it is common for some dinoflagellate species to produce a variety of diverse PKS related compounds, impeding our ability to link specific pathways with particular compounds.

The large numbers of PKS domains in dinoflagellates have been linked to the production of numerous undetected and/or uncharacterized polyketide molecules [81]. In the screening hypothesis model [81,125,229], it is suggested that organisms may have selected specific evolutionary traits to increase the probability of developing a compound with potent biomolecular activity—one that enhances the generation and retention of chemical diversity, while also reducing the fitness costs [95]. Hence, organisms may produce numerous biomolecules simultaneously as a cost-effective way of generating chemical diversity, therefore increasing the likelihood of producing a rare molecule with a useful biological activity [95,229,230]. Such compounds could be synthesized as by-products due to the inherent reactivity of chemical intermediates accompanying core catalytic mechanisms [231]. These models and working hypotheses represent a useful lens by which to view the complex array of both polyketide toxic compounds and PKS genes that have begun to be discovered using genetic techniques. The development of targeted approaches, combined with advances in chemical identification of these complex compounds, will allow us to address this issue and advance the discovery of the genetic basis and regulation of dinoflagellate toxins in the future.

## Figures and Tables

**Table 1:** Putative *sxt* genes in the dinoflagellates.

Gene	Function *	Dinoflagellate Species	Reported from Transcriptome	Reported from Genome
**Core sxt Genes**				
*sxtA (sxtA4)*	Aspartate aminotransferase	*Alexandrium australiense* *Alexandrium catenella Alexandrium minutum Alexandrium ostenfeldii* *Alexandrium pacificum* *Alexandrium tamarense Gymnodinium catenatum Pyrodinium bahamense*	na Yes [57,192] Yes [57] na Yes [190,192,193,195] No [181,192] Yes [190] Yes [190]	Yes [57,190] Yes [57,181,190] Yes [57,190] Yes [177] Yes [57,190] Yes [57]; No [181,190] Yes [57,197] na
*sxtG*	Amidinotransferase	*Alexandrium affine* *Alexandrium andersoni* *Alexandrium australiense* *Alexandrium catenella Alexandrium insuetum* *Alexandrium minutum Alexandrium pacificum* *Gymnodinium catenatum* *Pyrodinium bahamense*	No [191] No [191] Yes [191] Yes [57] No [191] Yes [57] Yes [191,192,193,195] Yes [190,192] Yes [190,192]	Yes [191] Yes [191] Yes [191] Yes [191] Yes [191] Yes [191] Yes [191] Yes [191] na
*sxtB*	Cytidine deaminase	*Alexandrium catenella* *Alexandrium minutum* *Alexandrium pacificum*	Yes [57] Yes [57] Yes [192,193,195]	na na na
*sxtD*	Sterole desaturase	*Alexandrium pacificum*	Yes [192,193,195]	na
*sxtS*	Phytanoyl-CoA dioxygenase	*Alexandrium catenella* *Alexandrium minutum* *Alexandrium pacificum* *Gymnodinium catenatum* *Pyrodinium bahamense*	No [57]; Yes [192] Yes [57] Yes [192] Yes [192] Yes [192]	na na na na na
*sxtU*	Alcohol dehydrogenase	*Alexandrium catenella* *Alexandrium minutum* *Alexandrium pacificum* *Pyrodinium bahamense*	Yes [57,192] Yes [57] Yes [192,193,195] Yes [192]	na na na na
*sxtH/T*	Phenylpropionate dioxygenase	*Alexandrium catenella* *Alexandrium minutum* *Alexandrium pacificum*	Yes [57,192] Yes [57] Yes [192,193]; Yes as H/T/DIOX [195]	na na na
*sxtI*	O-carbamoyl transferase	*Alexandrium catenella* *Alexandrium minutum* *Alexandrium pacificum* *Pyrodinium bahamense*	Yes [57] Yes [57] Yes [192,193,195] Yes [192]	na na na na
*sxtF/M*	Multidrug efflux protein	*Alexandrium catenella* *Alexandrium minutum* *Alexandrium pacificum*	Yes [57] Yes [57] Yes [192,193,195]	na na na
*sxtP*	STX binding protein	*Alexandrium catenella* *Alexandrium pacificum*	Yes [192] Yes [192,195]	na na
**Other sxt Genes**				
*sxtL*	GDSL lipase	*Alexandrium pacificum*	Yes [192]	na
*sxtN*	Sulfotransferase	*Alexandrium pacificum*	Yes [192]	na
*sxtO*	Adenylylsulfate kinase	*Alexandrium pacificum*	Yes [193,195]	na
*sxtR*	Acetyl CoA N-acyltransferase	*Alexandrium catenella* *Alexandrium minutum*	Yes [57] Yes [57]	na na
*sxtW*	Ferredoxin	*Alexandrium pacificum*	Yes [195]	na
*sxtX*	Cephalosporine hydroxylase	*Alexandrium pacificum* *Pyrodinium bahamense*	Yes [192,193,195] Yes [192]	na na
*sxtZ*	Histidine kinase	*Alexandrium pacificum*	Yes [193,195]	na
*sxtPER*	Permease	*Alexandrium pacificum*	Yes [195]	na
*sxtACT*	Acetylase	*Alexandrium pacificum*	Yes [195]	na

* Function inferred according to [185]. na: not available.
